# Using Evidence and Data to Design an Intervention in the Project Community Model for Fostering Health and Wellbeing Among Adolescent Mothers and Their Children

**DOI:** 10.3389/fpubh.2021.584575

**Published:** 2021-02-26

**Authors:** Nettie Dzabala, Mtisunge Kachingwe, Ibrahim Chikowe, Carol Chidandale, Lotte van der Haar

**Affiliations:** ^1^Young Women's Christian Association, Blantyre, Malawi; ^2^Pharmacy Department, College of Medicine, University of Malawi, Blantyre, Malawi; ^3^Utrecht Centre for Global Challenges, Utrecht School of Economics, Utrecht University, Utrecht, Netherlands

**Keywords:** implementation science, psychosocial, parental stress, resilience, violence, mother

## Abstract

In this paper, quantitative and qualitative measurements of maternal psychosocial wellbeing were utilized in three districts in Malawi that guided decision-making to increase the wellbeing of adolescent mothers and promote the healthy upbringing of their children. The 1-year design stage of the study relied on several sources of information: literature search, prior project implementation of similar projects, discussions with officials at the Malawi Department of Social Welfare, and observation visits in the targeted districts. The approaches for collecting data mentioned were triangulated for the development of a baseline survey. The baseline survey generated systematically collected data of the experiences and recalls as well as the missing data from the preliminary evaluation of the existing data. The baseline data gave the Young Women's Christian Association (YWCA) insight on the type of intervention required in order to give a greater and more holistic effect on the beneficiaries. We also discuss the lessons we learned as to whether the assumptions we had made at the onset were correct. If they were not correct, we explained the measures we took to correct the design or implementation of the project. Finally, the data provided benchmarks for project monitoring and evaluation.

## Introduction

Young motherhood is a common threat Malawian girls face, which has negative effects on their future as well as the children they bear. For example, resilience, self-esteem, parental stress, exposure to intimate partner violence, and child-mother interaction might be affected as a result of the young motherhood (1–4). In Malawi, 29% of adolescent girls aged 15–19 have given birth (5) and over half of adolescent mothers still remain at risk of becoming pregnant again due to non-use of contraceptives (6). Young mothers may also be amongst the most socially and economically disadvantaged women, thus bearing the brunt of gender-based factors. These include role restrictions, high unpaid workloads and gender-based violence that would also put them at higher risk of common perinatal mental disorders, especially in Low- and Middle-Income Countries (LMICs) (7).

This tendency of becoming pregnant early deprives a significant part of the population from acquiring knowledge and skills to become successful. Additionally, becoming pregnant early could pose significant risks for the psychosocial wellbeing of the young mothers and their children. Young mothers may suffer from depression, which is also a significant risk factor for decreased psychosocial wellbeing and development for their children (8). For example, mothers suffering from depression may have difficulties in engaging in activities that stimulate their children's' development (9), while happier mothers can positively influence the verbal and motor skills and socio-emotional behaviors of their children (10, 11). Stewart et al. (12), in their study of pregnant women and young mothers in Malawi, found rates of depression ranging between 10.7 and 21.1%. In another study, depressive symptoms were common among rural Malawian adolescents where 90% were above the traditional cut-off for screening of depression (13).

Despite the large population of young mothers in Malawi (5, 6), there is currently limited knowledge on psychosocial wellbeing of the young mothers. Insights into the psychological wellbeing of young mothers are crucial for the development and implementation of effective programs for adolescents, adolescent mothers, and their children. There is also limited data about how programs are planned, how the data for program planning is generated, and the challenges and solutions made to enable smooth achievement of the program outcomes. The objectives of this paper are to:

- Document the sources of information used for generation of data and benchmarks for planning, implementation, monitoring, and evaluating the project;
- Document the process of design and implementation of the intervention; and
- Assess the effect of the data and process on the project planning and implementation process.

The paper is structured as follows: First, we describe the methods used for data collection. Then, we describe how we used the data collection to design the intervention and finish by describing our learning's during that process.

## Methodology

### Study Context

Although the initial study design was based on reports and observations, systematically collected data was needed for the implementation of the project as well as development of the monitoring and evaluation benchmarks. In addition, since the observations and prior studies had been done at different times, in different places, and were based on different aims and methodologies, it was challenging to come up with a consensus or evidence-based interventions, much as the data provided a baseline for planning.

### Data Collection

This was a three-phased, retrospective, and prospective study, done in 1 year. Phase one involved the retrospective data collection method. This process included literature reviews that targeted studies on psychosocial wellbeing of adolescent mothers and nutritional programs that enhance children's health. We conducted a comprehensive literature search of published and unpublished (gray) literature to identify relevant studies of programs to support expectant and parenting teen mothers and fathers using the following keywords: young mothers or adolescent mothers in combination with any of the following words; psychosocial wellbeing, psychosocial support, intervention, intimate partner violence, mother child interaction, resilience, self-esteem, parental stress and evidence-based intervention. We searched in Google scholar, MEDLINE, Scopus, CINAHL, PsycINFO, and ERIC databases. We also searched the gray literature using Google Custom Search to create search queries to examine the entire content of selected relevant websites. Finally, to ensure a thorough literature search, we examined the reference lists of relevant review articles. To be eligible for the review, a study had to meet the following eligibility criteria. First, it had to be a study released between 1997 and December 2016. Second, it had to evaluate a program that supported teenage mothers and their children. To allow for a broad range of programs, the review included studies of programs delivered to expectant and parenting teens using any format, including individual or group sessions and programs taking place in any type of public, private, or institutional setting. Third, a study had to use a quasi-experimental design (QED) or a randomized controlled trial (RCT) impact design to allow conclusions about the causal effectiveness of the program in relation to a comparison condition. Studies could include clusters (such as schools) or individuals. Studies without a comparison group, such as descriptive analyses or pre-post designs, were not considered. Fourth, most of the mothers and fathers in the study sample had to be younger than 18 years of age at intake. Fifth, the study had to examine at least one measure in psychosocial wellbeing of teenage mothers and development of children born to teenage mothers. Finally, studies had to include an analytic sample larger than 30 participants across the treatment and comparison groups. Studies that focused on other areas of teen development and did not focus of psychosocial wellbeing of teenage mothers were excluded. Additionally, studies not published in English and studies published prior to 1997 were excluded. All studies that met the review eligibility criteria were assessed by two authors from the team. Authors assessed the quality and execution of the study research designs using a written protocol to determine whether the study findings were at risk of bias. If there were any disagreements between the two authors, a third author would then review the paper. The literature review produced a total of 347 articles that talked about the described key words out of which 124 met the inclusion criteria.

Phase two involved reviewing documents from previous projects that had been implemented in the study areas by the implementing organization, other organizations, implementation partners, as well as personal recalls or testimonies.

Phase three involved a prospective data collection process, which involved a baseline survey. In each site, 45 adolescent young mothers were recruited for a baseline study (135 in total). The baseline survey provided a needs assessment and further understanding of the situation on the ground. The survey collected data about the psychosocial wellbeing of the young mothers, and the many parameters that were revealed or signaled out in the studies and observation studies that the YWCAMW had instituted earlier. The study collected demographical data, resilience, self-esteem, parental stress, mother-child interaction, and nutritional as well as child growth parameters. This data was collected through meetings and interviews with randomly selected households and individuals. The meetings and interviews were structured through pretested structured demographic and health questionnaires.

### Data Needs Assessment

The project implementation needed various kinds of data. Mainly, Malawi data confirming the widely reported problems of young mothers worldwide, existing data for the development of benchmarks for planning, implementation, monitoring, and evaluation of the project, sample projects that have worked on this kind of intervention elsewhere both in the developed and developing countries and project adaptation approaches were sought after.

### Preliminary Visits and Observations

Prior studies that identified the mental health status of adolescent mothers were conducted in other parts of the world or parts of Malawi outside our projected study site. As such, observations may differ from place to place because of cultural and individual differences. The YWCA Malawi instituted a preliminary informal study in the identified study sites of Machinga, Mulanje, and Blantyre. Additionally, the Department of Social Welfare was visited to collect first hand data of the parameters of the project that would have been used for project monitoring and evaluation as well as finding out if there were other programs working in the same concept that would provide the needed data parameters (see Table 1). Cases reported frequently concern women and the many challenges they face in these districts for use in the execution of our project. Similar patterns reported in the studies, like high rates of teenage pregnancy and high rates of gender based violence, were also reported at the Department of Social Welfare.

**Table 1:** Overview of information needed, and whether the information was already covered through the literature review, previous projects, or the baseline study.

**Information needed**	**Literature review**	**Previous projects**	**Baseline study**
Prevalence of young motherhood	Yes	Yes	Yes
Challenges that young mothers face and their impact	Yes	Yes	Yes
Young mothers psychosocial wellbeing (resiliency levels, self-esteem, levels, parental stress levels, IPV)	No	No	Yes
Household diet and nutrition status	No	No	Yes
Cognitive development status of the children	No	No	Yes
Language and communication capability of children and their mothers	No	No	Yes
Physical growth status of the children	No	No	Yes
Proportion of mothers and children that need intervention	No	No	Yes
Factors that enhance early childhood development	Yes	Yes	Yes
Information on impact of interventions on young mothers and children	Yes	Yes	No

The project sites were chosen due to many cases of teenage pregnancies reported in local media that showed that they were among the districts with high populations and high teenage pregnancies and early marriages (14–16). The large numbers of teenage pregnancies in these areas were attributed to, among others, poverty, traditional practices, and low literacy levels. Previously reported data showed that a large number of young girls and young mothers were particularly vulnerable to these factors. Furthermore, the YWCAMW had witnessed similar trends during earlier work with young women in southern Malawi since 2006.

### Previous Projects Data and Experience

The YWCA carried out many projects in these areas emphasizing on Income Generating Activities (IGA) empowerment, and programs to enhance adult literacy. Prior to the activities done in Mulanje [one of the areas of the Grand Challenges Canada (GCC) project with funds from Norwegian agency for development cooperation (NORAD) RAD], we carried out a project in 2014, called “Building assets of girls between the ages of 15–24.” This project was aimed at addressing the sexual and reproductive health and rights (SRHR) of young women. The Mulanje area was chosen because, in Mulanje, only 5% of the girls who undergo primary school complete secondary school. Furthermore, there is a 20% HIV prevalence rate in girls aged between 15 and 19, 26% girls aged between 15 and 19 have already begun childbearing, 20% are already mothers, and an additional 6% are pregnant with their first child. Additionally, over half of the women in Malawi are married by age 18, 20% of the girls have experienced violence, and 71% of girls that experience violence are those who are or have been married. Overall, women are most expected to experience sexual violence between ages 15–19. These facts in the district of Mulanje compelled us to select this location to improve the SRHR situation. The other two sites showed similar statiistics and hence they were also chosen for the study.

## Results

The data sources were evaluated in terms of the amount of information they provided to meet the needs of the project and their effect on the project information that was needed for smooth implementation and evaluation of the project. Table 1 below shows the details for this analysis.

### Intervention Programs and Their Selection Criteria

As a result of the information gathered from the three data collection approaches, assumptions were made, and several interventional programs were designed.

### Assumptions Derived From the Data and Impact on Project Planning/Intervention

**Figure 1:**
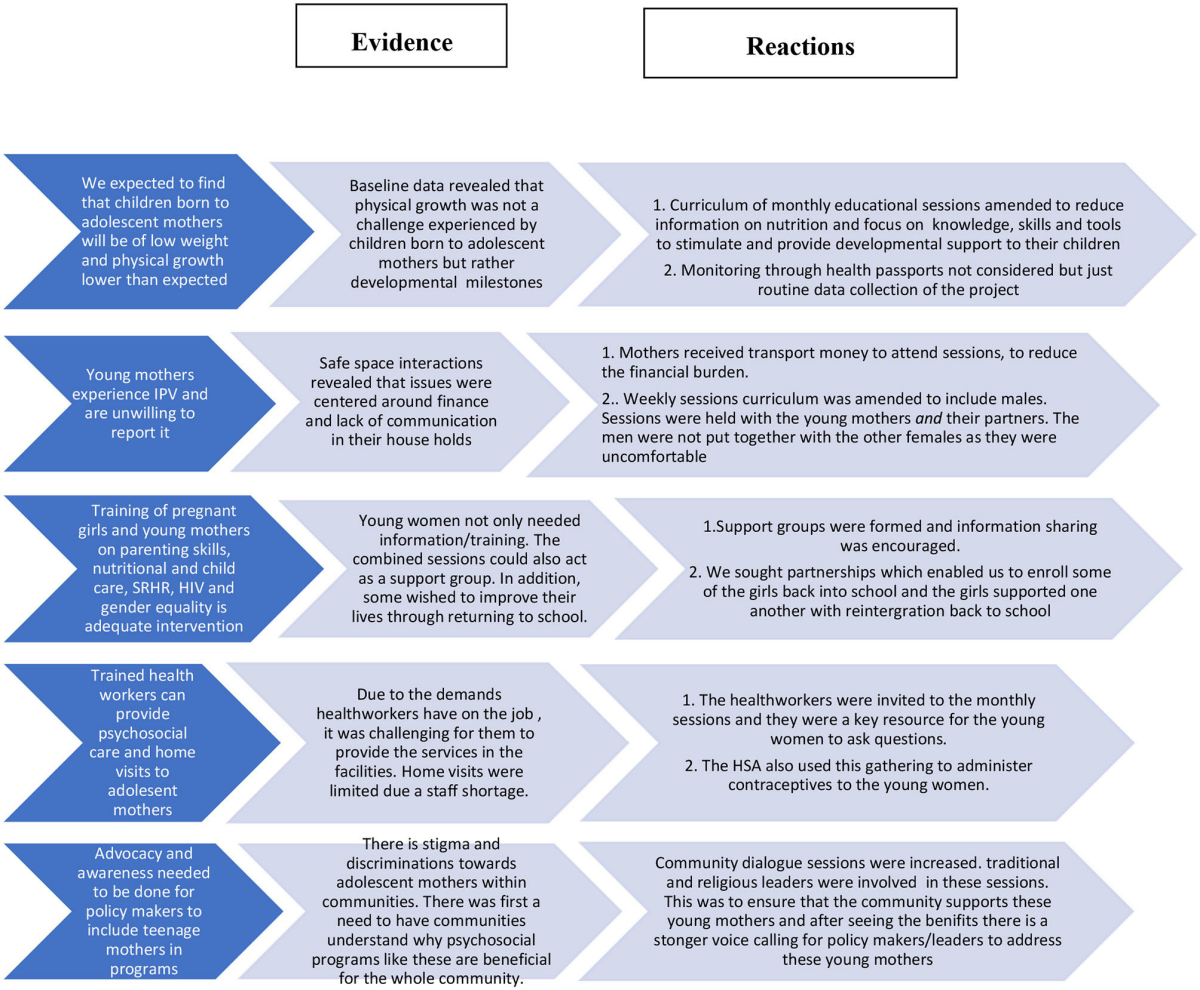
Movement from assumptions to evidence to reaction.

Based on baseline data, it was concluded that the young mothers were rooted in poverty. It was also found that young mothers had all the risk factors that make them vulnerable to Intimate Partner Violence (IPV): parental stress, low self-esteem, low resilience, and poor mother-infant interaction. Through the information gathered, it was found that mothers were not able to provide a balanced diet and meet the nutritional needs of their children. The young mothers were not in a position to help their children learn language and communication skills in addition to cognitive, motor, social and emotional capabilities. In sum, the young mothers were marginalized, and their children had poor early childhood development. The problems touched on emotional, physical, and brain development for both the mothers and their children. This knowledge necessitated the design of our project to include multiple domains; (1) psychosocial wellbeing of the mothers was approached through meetings where mothers created a safe space to share their daily challenges in their support group; (2) knowledge was addressed through having facilitators and inviting key stakeholders to teach mothers on various topics like brain development, hygiene and nutrition; (3) early childhood education and stimulation activities were provided for the children; and (4) advocacy in the community and districts were conducted so the adolescent mothers could have continued support. Figure 1 shows other assumptions that were made and how they evolved and guided project activities based on the data generated.

### Project Activities

The goal of the project was to improve early childhood development of children born to adolescent mothers and to improve psychosocial wellbeing of adolescent mothers in Malawi. It targeted the first 1,000 (2.5 years) days of a baby's life as it is a period of great brain development. To ensure early brain stimulation and that children were able to reach their developmental milestones appropriately, adolescent mothers were targeted to provide stimulating play activities and developmental support to their children. Adolescent mothers were empowered with parenting skills, nutritional care, child-care, and stimulation, so that they could develop strong bonds with their infants and improve the child's early brain development within the first 1,000 days of the baby's life. Feeding programs were included to supplement what the mothers were already doing at their homes to improve the nutrition of their children.

To help ensure the mother was in a good state to support her baby's development, Psychosocial Support (PSS) was provided for these adolescents to help them develop resilience and manage stress caused by poverty, forced marriage, gender-based violence, unwanted pregnancies, and social stigma. The project conducted monthly intervention sessions where adolescent mothers met to learn different PSS topics to improve their wellbeing and that of their babies. Development of positive parenting skills was critical as they supported the adolescent mothers to acquire and use appropriate skills in the upbringing of their children. More importantly, part of the parenting skills assists adolescent mothers in self-care, which also translates to one gaining the ability or understanding of how the same care given to one's child becomes helpful. The project also had a component of male engagement. This was done to enhance the role men play in the upbringing of children and also to reduce intimate partner violence through counseling.

### Activities to Stimulate Early Childhood Education

The activities were indirect with younger children and direct with the older children. For the younger children, the mothers were encouraged to engage in activities that stimulate the development of the children's brain through their mothers. The mothers learned the importance of playing with their children, speaking to their children, singing to their children and the importance of general care and cuddling. This helps in creating a stronger bond between the mothers (caregivers) and the child.

Older children were directly engaged. For instance, the children engaged in play with each other, singing, drawing, and/or playing with toys. All of these helped the child to get to use their entire body as much as possible while also stimulating the different parts of their brain.

### Sustainability of the Project

The project is being sustained as most children born to these adolescent mothers still participated in activities done at Community Child Care Centers (CBCCs) that were created during the project. These CBCCs are being run and coordinated by community members making use of the teachers who were trained during the project as well as learning materials that were purchased before. All CBCCs activities that are being done ensures continued growth and development of children. Through our network of volunteers and structures the women continue to meet. They contribute a small fee for the day care of their children and this goes to the upkeep of the children.

## Discussion

This paper describes the process of using qualitative and quantitative data to design an intervention targeted at improving young mothers and their children's wellbeing in Malawi. The data enabled the YWCA of Malawi to develop a program intervention that was holistic and tailored to the needs of the community and an effort to have a greater effect on the lives of the beneficiaries. The study enables the project to have a greater impact on the lives of the beneficiaries. The study highlights the importance of engaging communities in the design of an intervention and the importance of adapting the intervention to local parameters based on locally generated information.

The approach of using community experiences, consultations, and a baseline survey was also implemented in a community-based integrated nutrition research program by Oldewage-Theron et al. (17) in South Africa. This data collection approach helped ensure community participation and the intervention's sustainability. In Guatemala, a similar approach was used in the project implementation. A baseline study was conducted prior to project implementation for an integrated project involving agriculture, health and nutrition initiatives. The study generated important lessons that would be critical for effective implementation of projects and their monitoring and evaluation strategies such as information and programs to include in a project, how and where to get the information, required resources, communication channels, required knowledge and skillsets, and evaluation planning (18). The approaches reported in this paper are further supported by Peersman (19) who stated that data collection planning should start with review of existing data from standardized population based surveys like the Multiple Indicator Cluster Survey (MICS) conducted by UNICEF 2013–2014, Demographic and Health Survey (DHS), official statistics, program monitoring data, program records (including program descriptions, a theory of change, meeting minutes and others), formal policy documents, program implementation plans, progress reports and other studies.

The review of the existing data helps in the generation of data collection and analysis methods as well as prioritization of data gaps, feasibility of selected methods, data management approaches, obtaining quality data, developing an appropriate sampling strategy, conducting appropriate data analysis, and addressing ethical issues and practical limitations and implementing good practices. Furthermore, it is stated that baseline data for any intended project is vital for project implementation and evaluation, which helps in tracking changes over time. Where baseline data is unavailable, a systematic method of recall should be utilized if a formal survey is not feasible. Where the former is not feasible, although it may be biased but can be reduced with using survey tools. However, all data should be of sufficient quality, and there must be a robust relationship between different data sources (19).

The study's limitation included the fact that there was no statistically generated sample size due to the lack of baseline data for use in the calculation of a sample size. This limits the generalizability of the conclusions we will be able to draw for evaluation of the intervention. Secondly, the study was carried out in areas with similar cultures, which does not answer the question whether all cultures and societies would allow this type of work be done with its constituents due to the study design and implementation. However, the process of qualitative data collection specific to the Malawian context, e.g., through conversations with the Malawi Department of Social Welfare, is a process that can be used in any context.

The study's strengths included it speaking to an area of research that is lacking (using qualitative and quantitative evidence for effective intervention design), and the tools that we used were robust. The process of using evidence and data to design the intervention ensured that we had the adolescent mother and their communities' needs as factual and not assumed. Therefore, the interventions that were done were likely to produce positive result. This is shown in the paper “Assessing the impact of an intervention project by the Young women's Christian Association of Malawi on psychosocial wellbeing of adolescent mothers and their children in Malawi” by (20). It would be prudent for the policy akers to design their interventions based on data and evidence and not assumptions so that the babies born to these mothers have a chance at a better future and that would also bring about a positive outlook for the Malawi nation as a whole.

## Data Availability Statement

The raw data supporting the conclusions of this article will be made available by the authors, without undue reservation.

## Ethics Statement

The studies involving human participants were reviewed and approved by Ethical Approval and Consent to Participate Ethical approval was obtained from the College of Research and Ethics Committee (COMREC) of the University of Malawi with reference number P.05/17/2178. Written informed consent to participate in this study was provided by the participants' legal guardian/next of kin. Written informed consent was obtained from the individual(s), and minor(s)' legal guardian/next of kin, for the publication of any potentially identifiable images or data included in this article.

## Author Contributions

ND: abstract writing and manuscript writing. MK: methodology and manuscript writing. CC: methodology. IC: analysis of results, discussion, and manuscript writing. LH: review and editing of the manuscript. All authors revised and approved the final draft.

## Conflict of Interest

The authors declare that the research was conducted in the absence of any commercial or financial relationships that could be construed as a potential conflict of interest.
